# Effects of Oleacein, a New Epinutraceutical Bioproduct from Extra Virgin Olive Oil, in LPS-Activated Murine Immune Cells

**DOI:** 10.3390/ph15111338

**Published:** 2022-10-28

**Authors:** Rocío Muñoz-García, Marina Sánchez-Hidalgo, Tatiana Montoya, Manuel Alcarranza, Juan Ortega-Vidal, Joaquín Altarejos, Catalina Alarcón-de-la-Lastra

**Affiliations:** 1 Department of Pharmacology, Faculty of Pharmacy, Universidad de Sevilla, 41012 Sevilla, Spain; 2 Instituto de Biomedicina de Sevilla, IBiS, Universidad de Sevilla, HUVR, Junta de Andalucía, CSIC, 41013 Seville, Spain; 3 Department of Inorganic and Organic Chemistry, Faculty of Experimental Sciences, Campus de Excelencia Internacional Agroalimentario (ceiA3), Universidad de Jaén, 23071 Jaén, Spain

**Keywords:** epigenetic, histone, inflammation, immunity, macrophages, oleacein, olive oil, spleen cells

## Abstract

The present study was designed to evaluate the immunomodulatory effects of the secoiridoid from extra virgin olive oil, oleacein (OLA), deepening into the possible signaling pathways involved in LPS-activated murine peritoneal macrophages. Moreover, we have explored OLA-induced epigenetic changes in histone markers and related cytokine production in murine LPS-stimulated murine splenocytes. Murine cells were treated with OLA in the presence or absence of LPS (5 μg/mL) for 18 or 24 h. OLA modulated the oxidative stress and the inflammatory response produced by LPS stimulation in murine peritoneal macrophages, by the inhibition of pro-inflammatory cytokines (TNF-α, IL-6, IL-1β, IFN-γ, IL-17 and IL-18) and ROS production and the expression of pro-inflammatory enzymes such as iNOS, COX-2 and m-PGES1. These protective effects could be due to the activation of the Nrf-2/HO-1 axis and the inhibition of JAK/STAT, ERK and P38 MAPKs and inflammasome canonical and non-canonical signaling pathways. Moreover, OLA modulated epigenetic modifications throughout histone methylation deacetylation (H3K18ac) and (H3K9me3 and H3K27me) in LPS-activated spleen cells. In conclusion, our data present OLA as an interesting anti-inflammatory and antioxidant natural compound that is able to regulate histone epigenetic markers. Nevertheless, additional in vivo studies are required to further investigate the beneficial effects of this EVOO secoiridoid, which might be a promising epinutraceutical bioproduct for the management of immune-related inflammatory diseases.

## 1. Introduction

Macrophages are one of the most important components of the immune system and play a critical role in the inflammatory process. However, abnormal macrophage activation is involved in the pathogenesis of immune-inflammatory diseases. Specifically, an imbalance in the polarization of M1/M2 macrophages has been described as a key factor in the development of systemic lupus erythematosus [1]. Furthermore, macrophages are implicated in rheumatic diseases such as rheumatoid arthritis and osteoarthritis promoting synovitis and the destruction of cartilage and bone, characteristics of these diseases [2,3].

In activated macrophages, the Toll-like receptor 4 (TLR4) recognizes the pathogen-associated molecular pattern, bacterial lipopolysaccharide (LPS), leading macrophages to an inflammatory state and generating reactive oxygen species (ROS), Th1 and Th17 cytokines and chemokines. Furthermore, the expression of inflammatory-related enzymes, in particular, cyclooxygenase (COX)-2, microsomal prostaglandin E synthase (mPGES)-1, and inducible nitric oxide synthase (iNOS), which synthesize prostaglandin (PG)E2 and nitric oxide (NO), respectively, has been widely reported in LPS-stimulated macrophages [4,5]. The process of gene expression of these pro-inflammatory mediators involves multiple signaling cascades that culminate in the activation of transcription factors, mainly mitogen-activated protein kinases (MAPKs), nuclear transcription factor kappa B (NF-κB), mitogen-activated protein kinases (MAPKs), Janus kinase/signal transducer and transcription activator of transcription (JAK/STAT) or inflammasome activation [6].

In addition, the nuclear factor (erythroid-derived 2)-like 2 (Nrf-2) is a redox-sensitive transcription factor that regulates the expression of antioxidant enzymes such as heme oxygenase-1 (HO-1) [7]. This signaling pathway is repressed during the induction of activated macrophages through a regulative function on the activation of ROS, MAPKs, and inflammasome signaling pathways [8]. 

Dietary nutrients could regulate pathological processes through critical epigenetic mechanisms, modulating gene expression without altering the genetic code. In particular, extra virgin olive oil (EVOO) has exhibited anti-inflammatory activities in human macrophages by epigenetic mechanisms [9]. The beneficial effects of EVOO have been attributed to the high content of monounsaturated fatty acids, specifically oleic acid, and also a combination of minor phytochemicals, including polyphenols such as secoiridoids. Secoiridoids mainly represent the major group of phenolic compounds (>90%), particularly ligustroside and oleuropein aglycons and their derivatives oleocanthal and oleacein (OLA), respectively. In fact, during the EVOO extraction process, oleuropein is hydrolyzed by endogenous glycosylases to generate OLA [10].

Several studies have demonstrated the protective effects of OLA on the metabolic alterations presented in a high-fat diet-fed mice model, such as body weight gain, obesity, steatohepatitis, and insulin resistance [11,12]. In addition, OLA inhibited the formation of foam cells in human monocyte-derived macrophages [13] and prevented the destabilization of atherosclerotic plaque from hypertensive patients [14]. Additionally, this phenolic compound has shown anticancerous activity [15]. Singularly, OLA has exhibited anti-proliferative and anti-metastatic effects in myeloma multiple and SH-SY5Y human neuroblastoma cell lines [16,17]. Interestingly, OLA presented anti-inflammatory and antioxidant effects in a murine model of encephalomyelitis, improving the disease progression [18]. Moreover, OLA has been shown to be a potent antioxidant even more than its precursor oleuropein [19]. 

On the other hand, epigenetic changes such as histone modifications, DNA methylation, and non-coding RNA profiling have been described to play a significant role in the pathogenesis of autoimmune diseases. Immune system tolerance is regulated by histone modifications, preventing damage from an exacerbated inflammatory response. In this context, post-translational histone modifications could hold great therapeutic potential [20,21]. In fact, Lauterbach et al. have shown that macrophages redirect metabolic fluxes to foster histone acetylation in response to TLR4-LPS ligation in a time frame that is relevant for gene induction [22].

We have previously reported that LPS can promote the production of inflammatory cytokines in murine splenocytes and peritoneal macrophages suggesting that LPS stimulation disrupted H3 acetylation or methylation resulting in sustained IL-1β, IL-6, and IL-17 production [23,24,25]. Consequently, it could be considered that the use of both types of immune cells could provide a more complete view for a better comprehensive understanding of the potential effects of OLA on the immune-inflammatory response. In this context, prior studies by our research group have explored drug-induced epigenetic changes in histone markers (H3K9me3, H3K27me3, and H3K18ac) and cytokine-correlated production compared in spleen cells after LPS induction with relevant results production [25].

Given this background, the present study was designed to assess the antioxidant and anti-inflammatory effects of OLA in LPS-stimulated murine peritoneal macrophages. We attempted to characterize the action mechanisms underlying the possible beneficial effects. In addition, to explore the epigenetic mechanisms involved in the anti-inflammatory effects of this EVOO secoiridoid, we evaluated OLA-induced epigenetic changes in histone markers (H3K9me3, H3K27me3, and H3K18ac) and related cytokine levels in murine LPS-stimulated splenocytes.

## 2. Results

### 2.1. Isolation of OLA from Olive Oil

The amount of OLA required to carry out this work was obtained from a sample of olive oil of the cultivar Picual elaborated in the province of Jaen, Spain. The phenolic fraction was obtained by extraction of the oil with a mixture of methanol–water according to the International Olive Council protocol. The resulting dry extract (0.4% yield, with respect to oil) was chromatographed by the fast centrifugal partition chromatography (FCPC) technique yielding several fractions, one of them containing crude OLA, which was further chromatographed by semi-preparative high-performance liquid chromatography (HPLC) to give pure OLA (2.1% yield, with respect to phenolic fraction). The structure of this final sample of OLA was properly characterized by nuclear magnetic resonance (NMR) and polarimetry as (–)-oleacein as described in Section 4.1.

### 2.2. Antioxidant Activity of OLA

At first, the antioxidant effect of OLA was evaluated by inhibition of the 2,20-Azino-Bis (3-Ethylbenzothiazoline-6-Sulfonic Acid) (ABTS)+ radical. OLA presented a value of IC50 for ABTS radicals of 23.92 ± 3.96 μM. OLA showed similar antioxidant activity to the standard TROLOX, which presented IC50 values of 20.8 ± 3.81 μM.

### 2.3. Effects of OLA on Cell Viability

**Figure 1:**
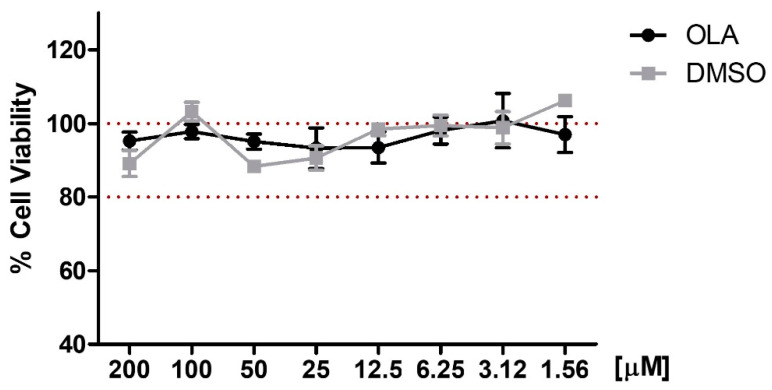
Effect of OLA on cell viability. Cells were treated with OLA (200–1.6 μM) for 18 h. Cell survival was expressed as the percentage of viability with respect to control untreated cells (100%). Data are represented as means ± SEM (*n* = 6).

We determined the effect of OLA on the viability of murine peritoneal macrophages by the sulforhodamine B (SRB) assay. After 18 h of OLA treatment, at concentrations of 200–1.56 μM, the viability of peritoneal macrophages was not significantly affected (Figure 1). In all doses the survival % was higher than 80%, taking as a reference the untreated control cells. In the same way, the vehicle dimethylsulfoxide (DMSO) did not alter cell viability.

### 2.4. Intracellular ROS, Nitrite Production, and iNOs Overexpression Are Inhibited by OLA in LPS-Stimulated Murine Peritoneal Macrophages

**Figure 2:**
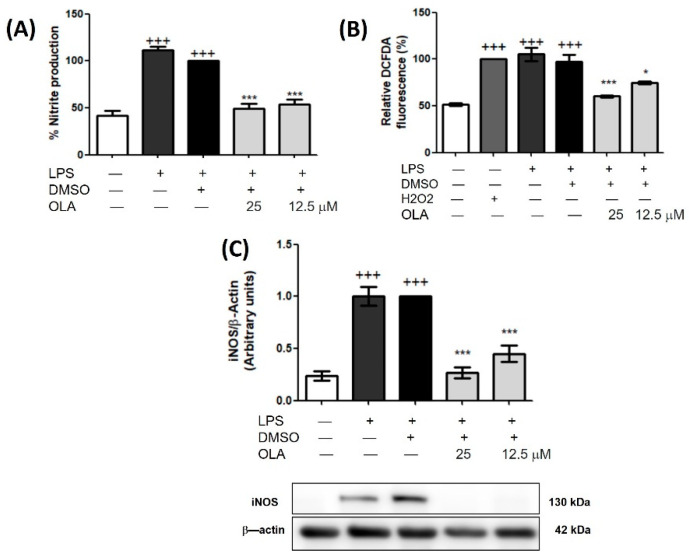
OLA downregulated ROS production, nitrites production, and iNOS protein expression in murine peritoneal macrophages. Cells were pretreated with different concentrations of OLA (25 and 12.5 μM) and then stimulated with LPS for 18 h. (**A**) Nitrite production; (**B**) ROS production; (**C**) Western blot analysis of iNOS protein expression. Densitometry was performed following normalization to the control (β-actin housekeeping gene). Data are represented as means ± SEM (*n* = 6). (+++) *p* < 0.001 vs. control cells (no stimulated). *** *p* < 0.001 vs. LPS-MSO-treated cells.

To know the effect of OLA on the inflammatory and oxidative response induced by LPS, we measured intracellular ROS, NO levels, and iNOS expression using 2′,7′–dichlorofluorescein diacetate (DCFDA), Griess, and Western blot assays, respectively, in LPS-activated murine peritoneal macrophages. The DCFA fluorescence signal was increased by LPS stimulation (+++ *p* < 0.001 vs. unstimulated cells). However, pretreatment with OLA was able to reduce ROS production (* *p*< 0.05, *** *p <* 0.001 vs. LPS-DMSO cells) (Figure 2B). This proves that OLA could have antioxidant activity.

Nitrites measurement was used as an indicator of NO production. Like the DCFDA signal, nitrites generation increased in the presence of LPS (+++ *p <* 0.001 vs. unstimulated cells). Nonetheless, pretreatment with OLA displayed a significant reduction of NO production in treated cells (*** *p <* 0.001 vs. stimulated cells). These data were related to the reduction in iNOS protein expression after OLA treatment (*** *p <* 0.001 vs. stimulated cells) (Figure 2A,C, respectively).

### 2.5. OLA Decreased LPS-Induced Secretion of Pro-Inflammatory Cytokines

**Figure 3:**
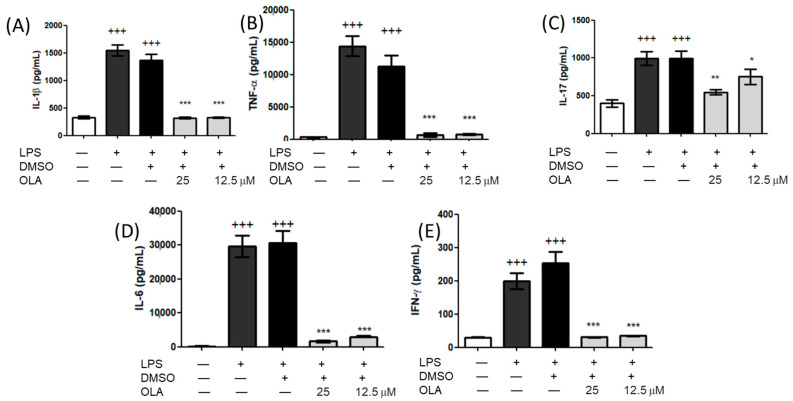
Effects of OLA on IL-1β (**A**), TNF-α (**B**), IL-17(**C**), IL-6 (**D**), and IFN-γ (**E**) levels. Cells were pretreated for 30 min(min) with OLA and LPS-stimulated (5 μg/mL) for 18 h. Control cells were also treated with DMSO (solvent control) and LPS or nontreated. Data are expressed as means ± SEM (*n* = 7). +++ *p* < 0.001 vs. control cells (no stimulated); * *p* < 0.05, ** *p* < 0.01, *** *p* < 0.001 vs. LPS−DMSO control-treated cells.

To assess whether OLA could inhibit the secretion of pro-inflammatory cytokines, specific ELISA assays were used to measure interleukin (IL)-1β, necrosis tumoral factor (TNF)-α, IL-17, IL-6 and interferon (IFN)-γ secretion levels in macrophages culture supernatant. After LPS stimulation for 18 h, the levels of IL-1β, TNF-α, IL-17, IL-6, and IFN-γ were significantly augmented (+++ *p* < 0.001 vs. unstimulated control cells). Nevertheless, exposure to OLA significantly decreased the secretion of all these cytokines compared to DMSO-LPS cells (* *p* < 0.05, ** *p* < 0.01, *** *p* < 0.001 vs. stimulated cells) (Figure 3A–E).

### 2.6. Inhibitory Effect of OLA on COX-2 and mPGES-1 Overexpression Induced by LPS

**Figure 4:**
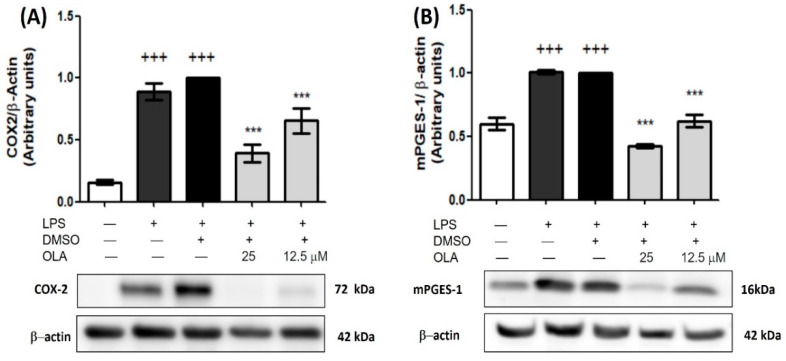
OLA reduced COX-2 and mPGES-1 overexpression in LPS-stimulated murine peritoneal macrophages. (**A**) Western blot analysis of COX-2 expression; (**B**) Western blot analysis of mPGES-1 expression. Cells were pretreated with OLA (25 and 12.5 μM) and incubated in absence or presence of LPS for 18 h. Densitometry was performed after normalization to the control (β-actin housekeeping gene). Data are represented as mean ± SEM (*n* = 6). +++ *p* < 0.001 vs. control cells (no stimulated); *** *p* < 0.001 vs. LPS−DMSO cells.

Next, in order to elucidate the anti-inflammatory activity of OLA, we analyzed the effects of OLA on the COX-2 and mPGES-1 markers. As shown in Figure 4, LPS stimulation increased COX-2 and mPGES-1 expression (+++ *p* < 0.001 vs. no stimulated cells). On the contrary, OLA pretreatment (25 and 12.5 μM) significantly decreased both COX-2 and mPGES-1protein expressions compared to LPS-DMSO cells (*** *p* < 0.001 vs. LPS-DMSO control cells) (Figure 4A,B).

### 2.7. OLA Upregulated Nrf-2/HO-1 Pathway Protein Expression

To evaluate whether OLA could regulate the antioxidant signaling pathway Nrf-2/HO-1, we determined the expression of Nrf-2 and HO-1 protein by Western blotting. Nrf-2 is a key transcription factor in the response to oxidative stress, which can regulate the expressions of antioxidant enzymes such as HO-1 [8].

**Figure 5:**
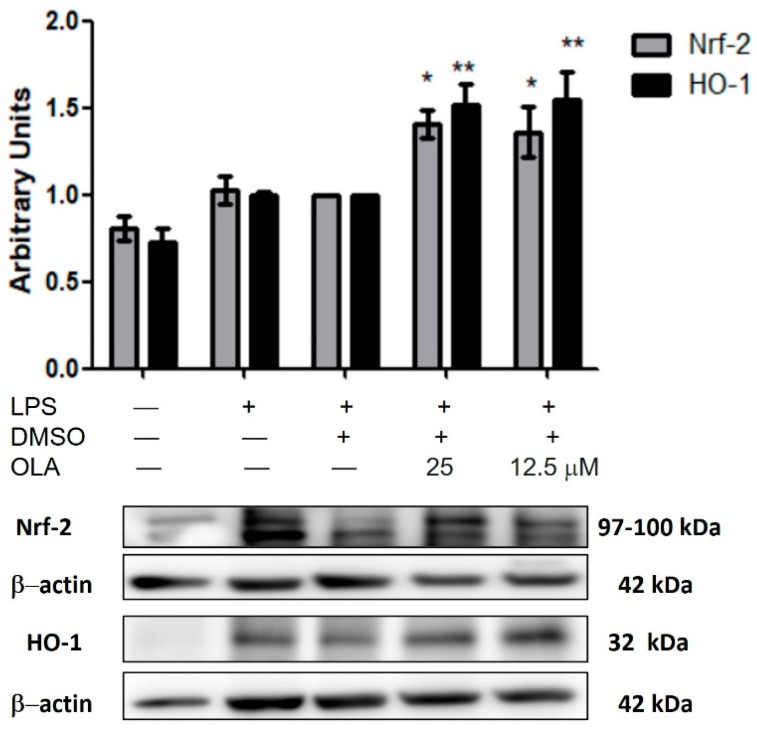
OLA upregulated Nrf-2/HO-1 signaling pathway in LPS-activated peritoneal macrophages. Murine cells were pretreated with OLA for 30 min, followed by LPS stimulation for 18 h. Nrf-2 and HO-1 protein levels were detected by Western blot. Densitometry was performed following normalization to the control (β-actin housekeeping gene). Data are represented as the means ± SEM (*n* = 6). * *p* < 0.05, ** *p* < 0.01 vs. LPS−DMSO-treated cells.

Treatment with OLA caused a significant increase in Nrf-2 and HO-1 expression compared to LPS-DMSO cells (* *p*< 0.05, ** *p* < 0.01 vs. LPS−DMSO-treated cells) (Figure 5). These results, along with ROS production downregulation could prove the potential role of OLA in the antioxidant response.

### 2.8. Effects of OLA on MAPKs Phosphorylation

**Figure 6:**
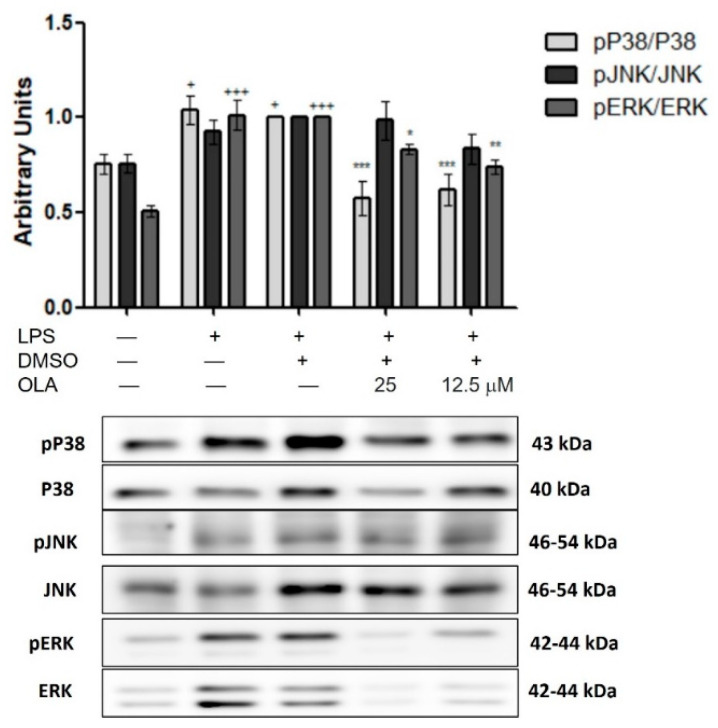
Effects of OLA on MAPKs activation in murine peritoneal macrophages. Cells were pretreated with OLA (25 and 12.5 μM) for 30 min and then incubated in presence or absence of LPS for 18 h. As controls, cells were also treated with DMSO (solvent control). Densitometry was performed after normalization to the control (P38, JNK, ERK housekeeping gene). Results are represented as means ± SEM (*n* = 6). + *p* < 0.05, +++ *p* < 0.001 vs. control cells (no stimulated); * *p* < 0.05, ** *p* < 0.01, *** *p* < 0.001 vs. LPS−DMSO-treated cells.

In addition, we assessed the role of OLA in MAPKs activation by Western blotting. Our results showed that OLA inhibited the phosphorylation of P38 and ERK induced by LPS stimulation (* *p* < 0.05, ** *p* < 0.01, *** *p* < 0.001 vs. LPS−DMSO control cells). Despite this fact, OLA was unable to significantly reduce JNK MAPKs phosphorylation after LPS treatment, as shown in Figure 6.

### 2.9. LPS-Induced JAK/STAT Pathway Activity Is Downregulated by OLA in Murine Peritoneal Macrophages

To go deeper into the possible molecular mechanism of OLA, we explored its potential role in JAK/STAT phosphorylation. The JAK/STAT signaling pathway participates in the inflammatory process to mediate immune responses. Usually, JAK2 is in the cytoplasm. Under cellular stress, it is phosphorylated and activated. Subsequently, STAT-3 is phosphorylated on tyrosine residues and translocated to the nucleus to bind DNA and moderate gene transcription [5,6].

**Figure 7:**
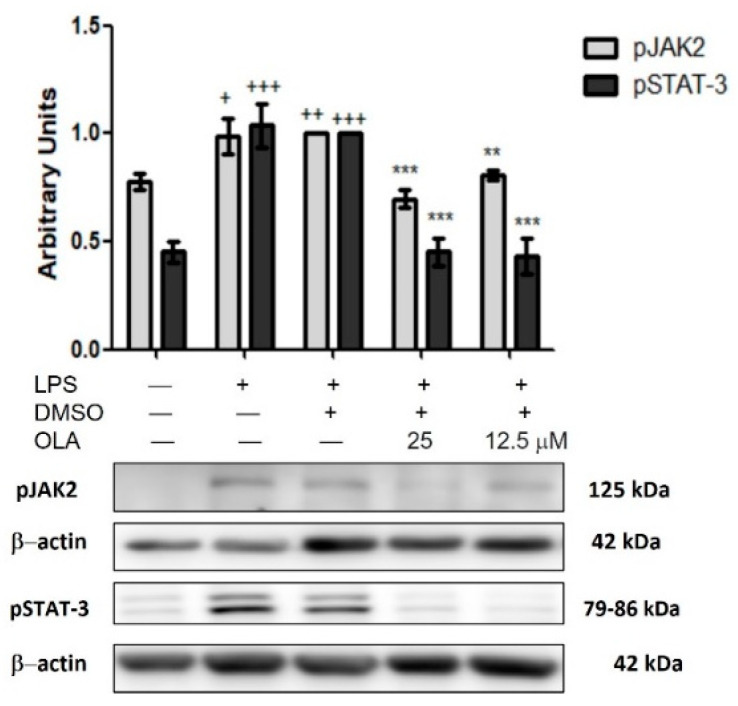
OLA treatment modulated JAK/STAT signaling pathway in LPS-stimulated murine peritoneal macrophages. Cells were untreated or treated with OLA (25 and 12.5 μM) for 18 h in presence or absence of LPS. Data shown are means ± SEM (*n* = 6). + *p* < 0.05, ++ *p* < 0.01, +++ *p* < 0.001 vs. control cells (no stimulated); ** *p* < 0.01, *** *p* < 0.001 vs. LPS−DMSO-treated cells.

We observed that LPS treatment significantly increased JAK2 and STAT-3 phosphorylation in peritoneal macrophages (+ *p* < 0.05, ++ *p* < 0.01, +++ *p* < 0.001 vs. non-stimulated cells) while in cells treated with OLA, JAK2 and STAT-3 phosphorylation was significantly downregulated (** *p* < 0.01, *** *p* < 0.001 vs. cells treated with LPS-DMSO) (Figure 7).

### 2.10. Effect of OLA in the Canonical and Non-Canonical Inflammasome Activation in LPS-Activated Murine Peritoneal Macrophages

“NOD-like” receptor (NLRP)-3 activation triggers an increase in the oligomerization of the adapter protein caspase recruitment domain (ASC). Consequently, ASC activates pro-caspase-1 in caspase-1, which is involved in the maturation of IL-1-β and IL-18 [26]. Hence, we analyzed the effects of OLA on canonical and non-canonical inflammasome activation using Western blot.

**Figure 8:**
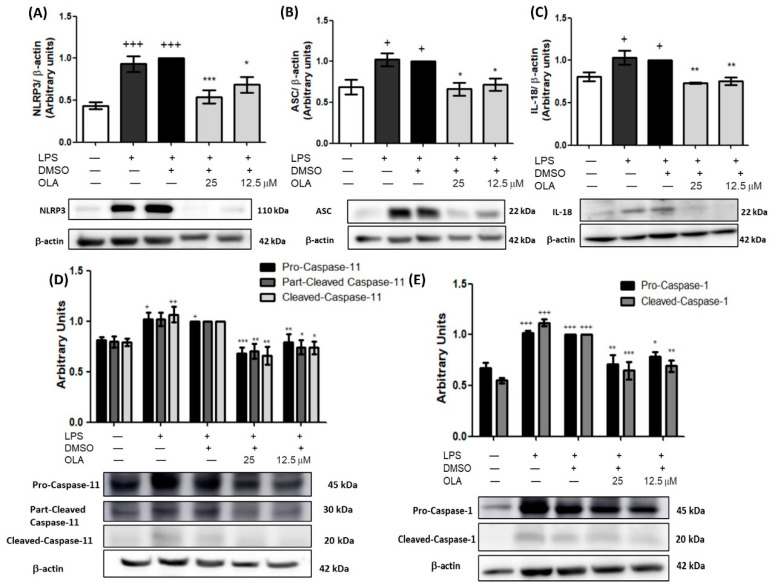
Inflammasome signaling pathway was downregulated by OLA in murine peritoneal macrophages. (**A**) NLRP3, (**B**) ASC, (**C**) IL-18, (**D**) pro-caspase-11, partially cleaved and cleaved, (**E**) pro-caspase-1 and cleaved-caspase-1. Protein expressions were analyzed by immunoblot. Murine peritoneal macrophages were pretreated for 30 min with OLA (25 and 12.5μM) followed by stimulation with 5 μg/mL LPS for 18 h. Densitometry was performed following normalization to the control (β-actin housekeeping gene). Data are represented as the means ± SEM (*n* = 6). + *p* < 0.05, ++ *p <* 0.01, +++ *p* < 0.001 vs. control cells (no stimulated); * *p* < 0.05, ** *p* < 0.01, *** *p* < 0.001 vs. LPS−DMSO-treated cells.

Our results showed that after LPS-stimulation there was an increase in NLRP3 and ASC protein expression compared to unstimulated cells, also, we observed parallel results in both forms of caspase-1 (+ *p* < 0.05, ++ *p* < 0.01, +++ *p* < 0.001 vs. no stimulated cells). However, in those cells treated with OLA, the expression of these proteins was negatively regulated (* *p* < 0.05, ** *p* < 0.01, *** *p* < 0.001 vs. LPS-DMSO treated cells). According to these results, we found that OLA reduced both IL-1β and IL-18 levels, in comparison with LPS-DMSO induced cells (** *p* < 0.01, *** *p* < 0.001 vs. LPS−DMSO-treated cells) (Figure 3A and Figure 8C, respectively).

On the other hand, we evaluated the impact of OLA on noncanonical inflammasome activation in LPS-induced murine peritoneal macrophages (Figure 8D). As expected, pro-, partially cleaved and cleaved-caspase-11 were upregulated after LPS stimulus (+ *p* < 0.05, ++ *p* < 0.01 vs. control cells). Nevertheless, OLA treatment could mitigate this activation (* *p* < 0.05, ** *p* < 0.01, *** *p* < 0.001 vs. LPS−DMSO-treated cells).

### 2.11. Effects of OLA on LPS-Induced IκB-α Degradation in Peritoneal Macrophages 

**Figure 9:**
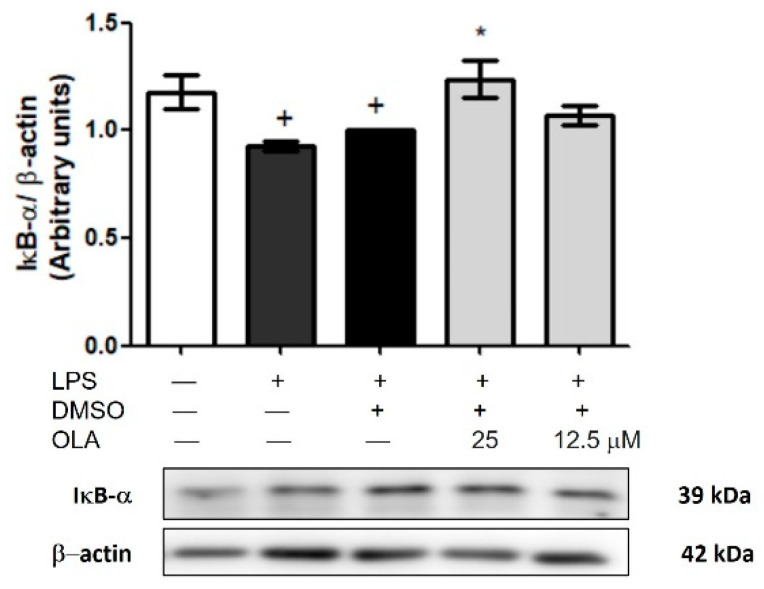
OLA pretreatment decreased IκB-α degradation in murine peritoneal isolated macrophages. Cells were pretreated with OLA (25 and 12.5 μM) and incubated in presence or absence of LPS for 18 h. Densitometry was performed following normalization to the control (β-actin housekeeping gene). Data are shown as means ± SEM (*n* = 6). + *p* < 0.05 vs. control cells (no stimulated); * *p* < 0.05 vs. LPS−DMSO-treated cells.

In addition, we evaluated the role of OLA in IκB-αdegradation in cytosolic fractions of murine peritoneal cells. After cellular stimulation, IκB-α is phosphorylated and degraded by the proteasome (+ *p <* 0.05 vs. no stimulated cells), leading to NF-κB signaling pathway activation [27]. Pretreatment with OLA at 25 μM prevented IκB-α degradation (* *p* < 0.05 vs. LPS-DMSO cells). However, at the dose of 12.5 μM this effect was not observed (Figure 9).

### 2.12. Epigenetic Histone Modifications by OLA in Spleen Cells

Finally, in order to explain the role of OLA in epigenetic changes, we studied whether this secoiridoid could induce post-translational modification in histone H3 and its correlated cytokines production.

**Figure 10:**
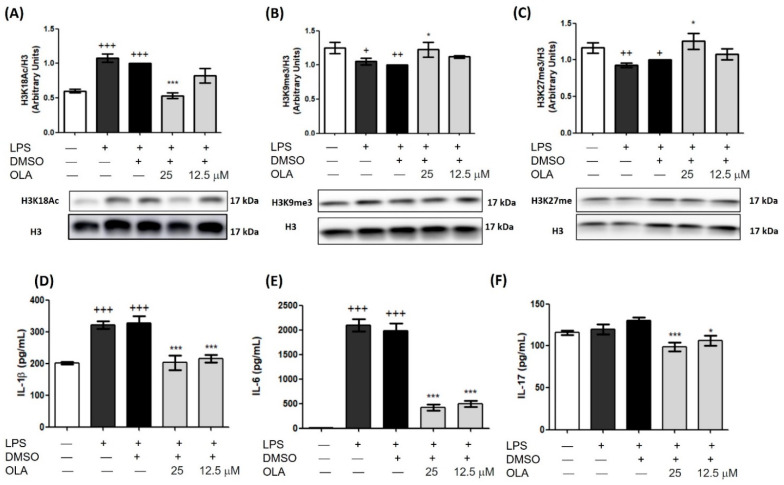
Modifications of histone acetylation (**A**) and methylation by OLA (**B**,**C**) and inhibition of the cytokine-related response (**D**–**F**). Spleen cells were treated with OLA (25 and 12.5 μM) and stimulated with LPS for 24 h. Histones were isolated from cells with acid extraction and were evaluated by Western blot. Densitometry was performed following normalization to the control (H3 housekeeping gene). Cytokine levels were measured in cell supernatants using ELISA assays. Results are presented as mean ± SEM of (*n* = 6). + *p* < 0.05, ++ *p* < 0.01, +++ *p* < 0.001 vs. control cells (no stimulated); * *p* < 0.05, *** *p* < 0.001 vs. LPS−DMSO-treated cells.

After LPS stimulation, H3K18ac levels were increased (+++ *p* < 0.001 vs. control cells). In cells treated with OLA (25 μM), we could appreciate a marked decrease in the acetylation level of Histone H3 (*** *p* < 0.001 vs. LPS-DMSO cells) (Figure 10A). However, we noticed the opposite effect on histone methylation levels. In OLA treated cells, we found a higher expression of methylated H3K9 and H3K27 while in the LPS cells the methylation of H3 was downregulated (Figure 10B,C) (+ *p* < 0.05, ++ *p* < 0.01, vs. control cells (no stimulated); * *p* < 0.05, ** *p* < 0.01, LPS−DMSO-treated cells).

Based on these results, we measured the production of cytokines that were associated with these epigenetic marks in their promoter regions. As expected, OLA reduced the LPS-induced overproduction of pro-inflammatory cytokines such as IL-1β, IL-6, and IL-17 in spleen cells (* *p* < 0.05, ** *p* < 0.01, *** *p* < 0.001 vs. LPS−DMSO-treated cells).

## 3. Discussion

The results obtained in our study have demonstrated, for the first time, that the natural secoiridoid of EVOO, OLA, reduced LPS-induced oxidative stress and acute inflammatory response in LPS-activated murine peritoneal macrophages. These data are in agreement with those of Czerwińska et al. [19] using non-cellular systems in vitro, as well as in human neutrophils and monocytes and with those of Gutiérrez-Miranda et al. in a murine model of experimental autoimmune encephalomyelitis [18].

The production of ROS by macrophages plays a critical role in the pathogenesis of inflammatory diseases. LPS induces its expression via several mechanisms such as the activation of nicotinamide adenine dinucleotide phosphate (NADPH) oxidase and the inhibition of antioxidant enzymes that participate in ROS clearance [28]. The disruption of intracellular redox balance leads to ROS-mediated damage-induced oxidative stress. After LPS stimulation, iNOS expression is induced and high levels of NO are produced, acting as an intracellular messenger that modulates ROS production [29]. In this context, Castejón et al. have previously reported the inhibitory action of oleuropein against LPS-induced ROS, NO, and iNOS production in murine macrophages [30]. However, our results showed that OLA treatment was not only able to decrease these oxidation-related mediators but also presented a higher reduction at lower concentrations (25 μM) in the same experimental conditions, according to Cirmi and colleagues [31]. Therefore, it should be noted that the potential antioxidant activity of OLA is even better than its pattern compound, oleuropein. On the other hand, data obtained in ABTS assay revealed non-cellular antioxidant activity of OLA similar to those observed in standard Trolox, in agreement with the results of Czerwinska et al. who confirmed the antioxidant activity of OLA, presenting it as a potent ROS scavenger [19]. 

This beneficial antioxidant ability can be attributed to the inhibition of the propagation chain during the oxidation process by the donation of radical hydrogen, which confers a marked free-scavenging ability associated with olive oil tree phenolic compounds’ beneficial properties [32].

Exposure to LPS is closely related to an imbalance of the cytokine network that induces the secretion of several pro-inflammatory Th1 and Th17 cytokines (IL-6, IL-1β, and TNF-α among others) [4,33,34]. In agreement with these observations, our data showed a marked increase in the expression of pro-inflammatory cytokines after LPS stimulation. Nonetheless, treatment with OLA significantly decreased the secretion of IL-6, IL-1β TNF-α, IFN-γ, and IL-17. Similar data have been described by Cirmi et al. on THP-1-derived macrophages [31]. Therefore, modulation of these pro-inflammatory biomarkers could represent a potential molecular target that is susceptible to OLA regulation.

In addition, LPS activation induces the overexpression of the inducible isoform of COX (COX-2) and mPGES-1, enzymes responsible for the overproduction of PGE2, which participates in the inflammatory process through the EP4 receptor activation [27,35,36]. Our data confirmed that LPS stimulation led to a significant increase in COX-2 and mPGES-1 expression, according to the studies mentioned above. By contrast, pretreatment with OLA reversed the action of LPS, diminishing the expression of both COX-2 and mPGES-1 in treated cells. In this sense, other studies have shown that OLA is able to inhibit COX-2 production in THP-1-derived macrophages [31] and human monocytes [37]. 

In addition, the multifunctional regulator Nrf-2 is considered a cytoprotective factor that regulates the expression of genes encoding antioxidant proteins, such as HO-1. This enzyme plays a cytoprotective role against oxidative stress and inflammation in activated macrophages, downregulating the expressions of iNOS, COX-2, NO, PGE2, TNF-α, IL-1β, IFN-γ, IL-6, among other pro-inflammatory mediators. Our study showed that the Nrf-2 / HO-1 signaling pathway was negatively regulated after LPS addition, according to our previous studies [25,27,36]. Other EVOO secoiridoids such as oleocanthal and oleuropein [30,35] presented similar effects. However, we provided novel evidence that OLA treatment inhibited the LPS-induced inflammatory response by significantly increasing the expression of Nrf-2/HO-1 proteins.

The cytoplasmic tyrosine kinase JAK2 is able to phosphorylate and dimerize the STAT-3 leading to its activation. Phosphorylated STAT-3 is translocated into the nucleus to bind DNA and moderate pro-inflammatory cytokines production [6]. Hence, we studied the effects of OLA on the JAK/STAT signaling pathway. Consistent with our prior studies, incubation with LPS promoted the phosphorylation of STAT-3 proteins in murine peritoneal macrophages [35,36]. In contrast, OLA was able to reduce this phosphorylation. Our results agree with those reported by Cirmi et al. in human neuroblastoma cells [16]. On the contrary, Filipek et al. demonstrated that OLA increased pSTAT-3 and pJAK2 levels in human monocyte-derived macrophages that promote changes in the macrophage phenotype from M1 to M2, thus inhibiting the development of atherosclerotic lesions [13]. Nevertheless, our results suggest that OLA could inhibit the expression of inflammatory cytokines by blocking the JAK/STAT pathway in LPS-activated macrophages.

As previously mentioned, LPS may stimulate MAPKs activation. MAPKs are a family of serine-threonine kinase enzymes that include extracellular signal-regulated kinases (ERKs-1 and -2), c-Jun N-terminal kinases (JNKs), and P38, which orchestrate the recruitment of gene transcription, protein biosynthesis, cell cycle control, apoptosis, and differentiation, allowing cells to respond to oxidative stress and inflammatory stimuli from their extracellular environment [38]. In addition, MAPKs may activate JAK/STAT inducing the expression of multiple genes that together regulate the inflammatory response [39]. Our data showed that after LPS incubation, JNK, P38, and ERK phosphorylation were increased, in agreement with recent studies [25,40]. On the other hand, OLA could modulate ERK and P38 phosphorylation. According to Lombardo et al., OLA regulated p-ERK expression in an in vivo high-fat-diet model [12]. Similar results have been obtained by other EVOO secoiridoids, in particular, oleocanthal [35] and oleuropein [30]; however, here we show for the first time that OLA treatment counteracted the effects of LPS by significantly decreasing the activation of both ERK and P38 MAPKs.

Emerging studies have shown that LPS-induced inflammation leads to the activation of the NLRP3 inflammasome in macrophages [25,35]. Inflammasomes are a group of multimeric cytosolic protein complexes that participate in the immune response and cell damage. The NLRP3 inflammasome is one of the most studied inflammasomes, comprising a sensor (NLRP3), an adaptor (ASC), and a zymogen (pro-caspase-1). Activation of the NLRP3 inflammasome leads to the recruitment of ASC, which is coupled with pro-caspase 1 to form a multimeric complex named the “canonical inflammasome”. This union causes caspase-1 maturation and, consequently, the activation of pro-IL-1β and pro-IL-18 in their mature forms inducing pyroptosis and inflammatory cell death [41]. On the other hand, non-canonical inflammasome activation is caused by caspase-11. In the presence of LPS, caspase-11 matures, resulting in potassium efflux and pyroptosis. The potassium efflux leads to the activation of NLRP3. Therefore, mature caspase-11 collaborates with the canonical NLRP3 inflammasome to induce the maturation of pro-IL-1 β and pro-IL-18 cytokines [42,43]. 

LPS-induced inflammation led to the activation of canonical and non-canonical inflammasome characterized by an increased expression of NLRP3, ASC, caspase-1, and caspase-11 which was accompanied by an increase in IL-18 and IL-1β production. Interestingly, our study showed, for the first time, the suppression by OLA of both canonical and non-canonical inflammasome pathways.

Thus, we provide evidence of the potential signaling pathways underlying the anti-inflammatory and antioxidant effects of OLA. OLA may block the inflammatory LPS-induced response in macrophages probably through regulation of the Nrf-2/HO-1, MAPKs, JAK/STAT and inflammasome signaling pathways. These data agree with our previous reports in which the olive secoiridoid, oleocanthal, and its metabolite were able to regulate the acute LPS-induced inflammatory response in murine peritoneal macrophages, controlling these inflammasome pathways [25,35]. 

It has been described that epigenetic alterations, including histone post-translational modifications, DNA methylation and miRNAs deregulation play an essential role in the inflammatory response. Usually, H3K9me3 and H3K27me3 methylated histones are associated with gene silencing due to the condensed structure of chromatin and reduced DNA accessibility [44]. Otherwise, H3 histone acetylation generally results in activated gene transcription. Thus, histone acetylation decreases the interactions between DNA and histones, leading to a relaxed chromatin conformation and promoting the accessibility of DNA to transcriptional factors [45]. Interestingly, several authors have described the possible implication of H3 post-translational modifications in pro-inflammatory cytokine production [46,47] and the role of histone methyltransferases in inflammasome activation, collaborating in the inflammasome-induced inflammatory response [48]. Furthermore, Sun et al. suggest that NLRP3 inflammasome activation depends on the level of histone H3 acetylation. In this regard, they showed that curcumin, a histone acetylation inhibitor, prevented NLRP3 activation [49].

Lately, OLA has been identified as a metabolo-epigenetic inhibitor of the mammalian target of rapamycin (mTOR) kinase and DNA methyltransferases (DNMTs) in cancer stem cells [50]. Additionally, this EVOO-related secoiridoid has been shown to inhibit lysine-specific histone demethylase 1A (LSD1) also known as lysine (K)-specific demethylase 1A (KDM1A) a central epigenetic regulator of metabolic reprogramming in obesity-associated diseases, neurological disorders, and cancer [51].

In addition, it has been proved that OLA is able to modulate the epigenome of melanoma cells by regulating the expression of genes and related regulatory microRNAs (miRNA) involved in anticancer and apoptotic activities [17,52]. Furthermore, OLA counteracts adipocyte inflammation by downregulating micro(mi)RNA-34a and miRNA-155 which are connected with the NF-κB pathway [53]. In this line, we have evaluated the effect of OLA on LPS-induced histone modifications in H3K9, H3K27, and H3K18 in spleen cells.

According to previous reports, increased H3K18ac and decreased H3K9me3 and H3K27me3 would be able to participate in the LPS induction of pro-inflammatory cytokine production [25]. Surprisingly, the treatment with OLA regulated these histone marks and subsequently controlled IL-6, IL-1β, and IL-17 production in spleen cells. To the best of our knowledge, this is the first report to show that OLA is a novel epigenetic regulator of proinflammatory cytokine production in immune cells.

## 4. Materials and Methods

### 4.1. Chemicals

#### 4.1.1. Reagents

Solvents used for extraction and fast centrifugal partition chromatography (FCPC), such as ethyl acetate (EtOAc), n-hexane (Hex), methanol (MeOH), and ethanol (EtOH) were of analytical grade and were purchased from VWR Chemicals (Prolabo^®^, Fontenay-sous-Bois, France). The water (H_2_O) used for chromatographic separations was of ultrapure grade produced by Milli-Q water (1.8 MΩ) equipment (Merck^®^, KGaA, Darmstadt, Germany). Acetonitrile (ACN) used for high-performance liquid chromatography (HPLC) and chloroform (CHCl3) used to determine optical rotation were of HPLC grade and were purchased from VWR^®^ (Madrid, Spain). Deuterated chloroform (CDCl_3_) was used to prepare solutions of the isolated compound for nuclear magnetic resonance (NMR) analyses and purchased from VWR^®^ (Madrid, Spain). Acetic acid (AcOH) used for HPLC was purchased from VWR^®^ (Madrid, Spain). 

#### 4.1.2. Instruments

HPLC analyses were performed on a Waters HPLC, equipped with a C18 reversed-phase Spherisorb ODS-2 column, 250 × 3 mm i.d, 5 μm (Waters Chromatography Division^®^, Mildford, MA, USA), and a photodiode array detector. Fractionation of phenolic olive oil extract was carried out on a FCPC-200^®^ instrument (Kromaton Technologies^®^, Angers, France) that is fitted with a rotor and total column capacity of 200 mL. Rotation can be adjusted from 0 to 2000 rpm. The solvent was pumped by an Alltech 627 isocratic pump (Alltech Associates^®^, Deerfield, IL, USA). The sample was injected into the FCPC column with a 3725i-038 manual injector (Rheodyne^®^, Cotati, CA, USA) equipped with a 10 mL sample loop. The instrument is equipped with a UV–Vis Linear UVIS 200 detector (Linear Instrument Co.^®^, Reno, NV, USA) set at 280 nm.

Purification of (–)-oleacein (OLA) from EVOO was performed on a Shimadzu semi-preparative HPLC system (Shidmadzu Scientific Instruments^®^, Columbia, MD) equipped with a C18 reversed-phase Spherisorb ODS-2 semi-preparative column (Waters), 250 × 10 mm i.d, 5 μm, and a photodiode array detector.

Proton nuclear magnetic resonance (1H NMR) and carbon nuclear magnetic resonance (13C NMR) spectra to elucidate the structure of OLA were recorded on a Bruker Avance DPX 400 spectrometer (Bruker Daltonic GmbH^®^, Rheinstetten, Germany) at 400 and 100 MHz, respectively, using a solution of OLA in CDCl3 with a drop of tetramethylsilane (TMS) as internal reference. Coupling constants (J) are provided in hertz (Hz) and the multiplicities of signals are reported using the following abbreviations: broad singlet (brs), doublet (d), doublet of doublets (dd), doublet of doublet of doublets (ddd), quadruplet (q) and multiplet (m).

A Jasco P-200 automatic polarimeter (Jasco Analytical Instrument^®^, Easton, MD, USA) was used to measure optical rotation (α) of OLA, in chloroform, in order to calculate its specific rotation value ([α]D).

#### 4.1.3. Isolation and Identification of (–)-Oleacein (OLA)

A sample of olive oil (300 g), obtained from ripe olives and provided by Drs. Francisco Espínola and Manuel Moya (Department of Chemical Engineering, University of Jaén, Spain), was extracted using the experimental procedure described by the International Olive Council (IOC, 2017) as reported by us in Diez-Bello et al. [54]. The resulting dry extract (1.5 g) was chromatographed with the FCPC instrument using a solvent system composed of Hex:AcOEt:EtOH:H2O (2:3:2:3, *v*/*v*/*v*/*v*). Seventeen fractions were collected and monitored by analytical HPLC using a step gradient with mixtures of MeOH/ACN (1:1, *v*/*v*, solvent A) and H2O/AcOH (99.8:0.2, *v*/*v*, solvent B) at a flow rate of 0.7 mL/min (min). The gradient program consisted of a linear gradient from 4 to 50% A in 40 min; a linear gradient from 50 to 60% A in 5 min; a linear gradient from 60 to 100% in 15 min; 100% A for 10 min; and other 12 min to return to the initial conditions. As a result, 200 mg of crude OLA was obtained, which was re-purified by semi-preparative HPLC using as solvent ACN/AcOH (99.8:0.2, *v*/*v*, solvent A) and H2O/AcOH (99.8:0.2, *v*/*v*, solvent B) at a flow rate of 5 mL/min. The separation was carried out with a linear gradient from 20 to 25% A. Finally, pure OLA (32 mg) was obtained and structurally characterized by ^1^H-NMR and ^13^C-NMR (Figures S2 and S3), and optical rotation; [α]D = −4,5 (c = 1.0,CHCl3). The spectroscopy data of this compound agreed with those reported in the literature for OLA [55]. 

### 4.2. Animals

Female Swiss mice (20–30 g) were provided by m the Animal Production Center of the University of Seville (Seville, Spain). Mice (8–10-week-old) were randomly distributed in cages and maintained under constant conditions of temperature (20–25 °C) and humidity (40–60%), a 12 h light/dark cycle, and ad libitum standard rodent chow (PanlabA04, Panlab^®^, Seville, Spain) and free water access.

All experiments were performed in the Faculty of Pharmacy Animal Laboratory Center (University of Seville, Spain) according to the Guidelines of the European Union regarding animal experimentation (Directive of the European Counsel 2012/707/EU) and following a protocol approved by the Animal Ethics Committee of the University of Seville (CEEA-US 2018-11/2 and CEEA-US 2019-6) and by the Consejeria de Agricultura, Pesca y Desarrollo (Junta de Andalucía, 23/07/2018/119), according to the RD 53/1 February 2013.

### 4.3. Isolation and Culture of Murine Peritoneal Macrophages and Spleen Cells

Peritoneal macrophages were isolated following the protocol described by Aparicio-Soto et al. [27]. Mice were injected with 1 mL of sterile thioglycolate medium (3.8% *w*/*v*) (BD Difco^®^, Le Pont de Claix, France). After 72 h, cells were harvested by peritoneal washes with sterile ice-cold phosphate-buffered saline (PBS). Macrophages were centrifugated and resuspended in RPMI1640 supplemented with 10% fetal bovine serum, penicillin, and streptomycin. Then, the cells were seeded in culture plates (1 × 10^6^ cells/mL) for 2 h at 37 °C in a 5% humidified atmosphere. Next, the medium was changed to remove non-adherent cells. Fresh RPMI1640 medium without FBS containing OLA treatment (25 and 12.5 μM OLA) was added. After 30 min, cells were stimulated with 5 μg/mL of LPS from Escherichia coli [27,56] (Sigma-Aldrich^®^, St. Louis, MO, USA) for 18 h. Finally, supernatants and cell samples were collected and stored at −80 °C.

Additionally, spleens were collected, crushed, and passed through a nylon cell strainer (70 μm) (BD Falcon^®^, USA) to prepare a single-cell suspension. Cells were cultured in supplemented RPMI1640 medium (10% fetal calf serum, 1% penicillin and streptomycin, 2 mM glutamine, 1 mM sodium pyruvate, and 50μM 2-mercaptoethanol). Splenocytes were pelleted and resuspended in red blood lysis buffer (BD^®^, Biosciences, Bloomfield Hills, MI, USA) to remove erythrocytes. The murine cells were then washed with PBS. Cells were cultured in 24-well plates at a final concentration of 3 × 10^6^ cells/mL at 37 °C in humidified air with 5% CO_2_ for 24 h in presence or absence of OLA (25 and 12.5μM) and LPS (5 μg/mL). After incubation, cells were centrifugated, and pellets and supernatants were collected and stored at −80 °C until ELISA assays and histone extraction. 

### 4.4. ABTS Test

To perform the ABTS test, we have followed the method described by Re et al. [57], using TROLOX (6-hydroxy 2,5,7,8-tetramethylchroman-2-carboxylic acid) as a positive antioxidant pattern.

Potassium persulfate and ABTS were solved in water until a final concentration of 2.45 and 7 mM, respectively. This solution was stood in darkness for at least 16 h to produce de radical ABTS+. The ABTS+ radical solution was diluted with absolute ethanol to an absorbance of 1.00 at 734 nm. OLA and Trolox samples (final concentrations 200–1.6 μM) were mixed with 70 μL of absolute ethanol and 100 μL of ABTS+ diluted solution. The absorbance was read at 734 nm for 6 min after mixing.

The results were expressed as IC50, the concentration (μM) of the compound that was able to inhibit 50% of ABTS+ radicals. All determinations were carried out in triplicate.

### 4.5. Cell Viability

Cells were seeded in 96-well plates (1 × 10^5^ cells/well) and incubated in the presence or absence of OLA (200–1.56 μM) for 18 h. Cell viability was measured using the SRB assay. After incubation, adherent cell cultures were fixed by adding 50 μL of 50% (*w*/*v*) cold trichloroacetic acid (Sigma–Aldrich^®^, St. Louis, MO, USA) and incubated for 60 min at 4 °C. The supernatant was discarded, and the plates were washed 5 times with distilled water. An amount of 50 μL of SRB (Sigma–Aldrich^®^, St. Louis, MO, USA) solution (0.4% *w*/*v*) in 1% acetic acid (Panreac^®^, Barcelona, Spain) was added to each well and incubated for 30 min in darkness at room temperature. Plates were washed 5 times with 1% acetic acid and air-dried. Then, 100 μL/well of 10 mmol/L Tris base, pH 10.5 (Sigma–Aldrich^®^, St. Louis, MO, USA) was added and the absorbance of each well was read on a multiwell plate reader (Biotek^®^, Bad Friedrichshall, Germany) at 492 nm. At last, the cell survival was measured as the percentage of absorbance compared with that obtained in non-treated cells (control cells). We also studied the toxicity of dimethyl sulfoxide (DMSO) which was used as a vehicle to dissolve OLA. 

### 4.6. Measurement of Nitrite Production

Cells were seeded in 24-well plates (1 × 10^6^ cells/mL) and treated with different concentrations of OLA (25 and 12.5 μM). After 30 min, peritoneal macrophages were stimulated with LPS for 18 h. Nitrites levels were determined by Griess reaction (Sigma-Aldrich^®^, St. Louis, MO, USA) through extrapolation from a standard curve with sodium nitrite 22. Absorbance at 540 nm was measured using an ELISA reader (BioTek^®^, Bad Friedrichshall, Germany) and represented as a percentage compared with LPS-DMSO cells.

### 4.7. DCFDA Cellular Reactive Oxygen Species Detection

Intracellular ROS were detected using a DCFDA assay kit (Abcam^®^, Cambridge, UK) according to the manufacturer’s instructions. The 2.5 × 10^4^ cells/well were seeded on a 96-well black plate and LPS-stimulated in presence or absence of OLA pretreatment (25 and 12.5 μM). DCFDA (25 μM) was added to each well at 37 °C for 45 min. The excitation and emission wavelengths (485 and 535 nm, respectively), were read using a fluorescence microplate reader (Biotek^®^, Bad Friedrichshall, Germany). We used H_2_O_2_ (Sigma-Aldrich^®^, Barcelona, Spain) as a positive pro-oxidant control (100% intracellular ROS production). 

### 4.8. Determination of Pro-Inflammatory Cytokines by Enzyme-Linked ImmunoSorbent Assay

Pro-inflammatory cytokines levels were measured on culture supernatants using IL-1β, TNF-α (R&D System^®^, Minneapolis, MN, USA), IL-17 (Peprotech^®^, London, UK), IL-6, and interferon (IFN)-γ (Diaclone^®^, Besancon Cedex, France) ELISA kits, according to manufacturer’s instructions. 

### 4.9. Histone Extraction

Histones were extracted using an acid extraction protocol [58] with modifications. Spleen cells were washed twice with PBS, resuspended in lysis buffer (10 mM Tris pH 6.5, 50 mM sodium bisulfite, 10 nM MgCl2, 8.6% sucrose, 1% Triton X-100) and incubated for 15 min at 4 °C. Then, cells were centrifuged at 3500× *g* rpm for 10 min at 4 °C and rewashed with lysis buffer. The supernatant was discarded and Tris-EDTA buffer (10 mM Tris pH 7.4 and 13 mM EDTA) was added to the samples. The pelleted nuclei were resuspended with acid sulfuric 0.2M. After, at least, 1 h of incubation, samples were centrifuged at 15,000× *g* rpm for 1 h at 4 °C. Supernatants were collected and incubated overnight at −20 °C with acetone. The samples were centrifuged at 15,000× *g* rpm for 10 min at 4 °C, pellets were diluted with H2O and the protein content was measured according to Bradford’s method [59]. 

### 4.10. Isolation of Proteins and Western Blot Analysis

Cells were lysed in ice-cold PBS containing a cocktail of protease and phosphatase inhibitors to isolate proteins. Protein concentration was determined according to the Bradford method [59] using γ-globulin as a standard.

Aliquots of 15 μg of protein were separated on 10% or 15% acrylamide gel by sodium-dodecyl sulfate polyacrylamide gel electrophoresis, transferred to a nitrocellulose membrane, and incubated overnight at 4 °C with specific primary antibodies against mPGES-1, IL-18 (Abcam^®^, Cambridge, UK), COX-2, iNOS, pJNK, JNK, P38, pP38, pERK1/2, ERK ½, Nrf-2, pSTAT-3, pJAK2, NLRP3, ASC, H3K18ac, H3K27me3, H3K9me3, H3 (Cell Signaling^®^, Danvers, MA, USA), HO-1 (Henzo^®^, Madrid, Spain), caspase-1 and caspase-11 (Novus Biologicals^®^, Littleton, CO, USA). Nitrocellulose membranes were incubated for 2 h in a blocking solution with antirabbit horseradish peroxidase-labeled (HRP) secondary antibody (Cell Signaling^®^, Danvers, MA, USA) or antimouse HRP secondary antibody (Dako^®^, Atlanta, GA, USA). β-Actin primary antibody (Abcam^®^, Cambridge, UK) was employed to demonstrate equal loading. The immunosignals were captured using Amersham Imager 600 from GE Healthcare^®^ (Buckinghamshire, UK) and analyzed and quantified by Image Processing and Analysis in Java (ImageJ^®^, Softonic) and expressed comparing with the DMSO-LPS treated cells.

### 4.11. Statistical Analysis

All values in the figures and text are expressed as arithmetic means ± standard error (SEM). Data were evaluated with GraphPad Prism_Version 5.01 software. The statistical significance of any difference in each parameter among the groups was evaluated by one-way analysis of variance (ANOVA), using Tukey–Kramer multiple comparisons test as post hoc test. *p* values of <0.05 were considered statistically significant. 

## 5. Conclusions

In conclusion, our study proves, for the first time, that OLA was able to modulate oxidative stress and the acute inflammatory response produced by LPS stimulation in murine peritoneal macrophages, by inhibiting the production of ROS, pro-inflammatory cytokines (TNF-α, IL-6, IL-1β, IFN-γ, IL-17, and IL-18) and pro-inflammatory enzymes such as iNOS, COX-2, and mPGES-1. These protective effects could be due to the activation of the Nrf-2/HO-1 axis and inhibition of JAK/STAT, ERK, and P38 MAPKs and inflammasome canonical and non-canonical signaling pathways. Moreover, OLA modulated epigenetic modifications through histone methylation (H3K9me3 adnH3K27me) and deacetylation (H3K18ac) in LPS-activated spleen cells. Despite these promising results, further in vivo studies would be necessary to explore the immunomodulatory effects of OLA, which could be a novel epinutraceutical bioproduct, useful in the management of immuno-inflammatory pathologies.

## Data Availability

Data are contained within the article.

## Supplementary Materials

The following supporting information can be downloaded at: https://www.mdpi.com/article/10.3390/ph15111338/s1, Figure S1: (–)-Oleacein chemical structure; Figure S2: ^1^H NMR of (–)-oleacein in deuterated chloroform; Figure S3: ^13^C NMR (bottom) and Dept-135 (superior) of (–)-oleacein in deuterated chloroform.

## References

[1] Funes S.C., Rios M., Escobar-Vera J., Kalergis A.M. (2018). Implications of Macrophage Polarization in Autoimmunity. Immunology.

[2] Griffin T.M., Scanzello C.R. (2019). Innate Inflamation and Synovial Macrophages in osteoarthritis Pathophysiology. Clin. Exp. Rheumatol..

[3] Tu J., Wang X., Gong X., Hong W., Han D., Fang Y., Guo Y., Wei W. (2020). Synovial Macrophages in Rheumatoid Arthritis: The Past, Present, and Future. Mediat. Inflamm..

[4] Jeong Y.H., Oh Y.C., Cho W.K., Yim N.H., Ma J.Y. (2019). Hoveniae Semen Seu Fructus Ethanol Extract Exhibits Anti-Inflammatory Activity via MAPK, AP-1, and STAT Signaling Pathways in LPS-Stimulated RAW 264.7 and Mouse Peritoneal Macrophages. Mediat. Inflamm..

[5] Li R., Hong P., Zheng X. (2019). β-Carotene Attenuates Lipopolysaccharide-Induced Inflammation via Inhibition of the NF-ΚB, JAK2/STAT3 and JNK/P38 MAPK Signaling Pathways in Macrophages. Anim. Sci. J..

[6] Huang Y.P., Chen D.R., Lin W.J., Lin Y.H., Chen J.Y., Kuo Y.H., Chung J.G., Hsia T.C., Hsieh W.T. (2021). Ergosta-7,9(11),22-Trien-3β-Ol Attenuates Inflammatory Responses via Inhibiting Mapk/Ap-1 Induced Il-6/Jak/Stat Pathways and Activating Nrf2/Ho-1 Signaling in Lps-Stimulated Macrophage-like Cells. Antioxidants.

[7] Zhang H., Yuan B., Huang H., Qu S., Yang S., Zeng Z. (2018). Gastrodin Induced HO-1 and Nrf2 up-Regulation to Alleviate H2O2-Induced Oxidative Stress in Mouse Liver Sinusoidal Endothelial Cells through P38 MAPK Phosphorylation. Braz. J. Med. Biol. Res..

[8] Lim D.W., Choi H.J., Park S.D., Kim H., Yu G.R., Kim J.E., Park W.H. (2020). Activation of the Nrf2/HO-1 Pathway by Amomum Villosum Extract Suppresses LPS-Induced Oxidative Stress in Vitro and Ex Vivo. Evid.-Based Complement. Altern. Med..

[9] Bordoni L., Fedeli D., Fiorini D., Gabbianelli R. (2020). Extra Virgin Olive Oil and Nigella Sativa Oil Produced in Central Italy: A Comparison of the Nutrigenomic Effects of Two Mediterranean Oils in a Low-Grade Inflammation Model. Antioxidants.

[10] Vierhuis E., Servili M., Baldioli M., Schols H.A., Voragen A.G.J., Montedoro G. (2001). Effect of Enzyme Treatment during Mechanical Extraction of Olive Oil on Phenolic Compounds and Polysaccharides. J. Agric. Food Chem..

[11] Lepore S.M., Maggisano V., Bulotta S., Mignogna C., Arcidiacono B., Procopio A., Brunetti A., Russo D., Celano M. (2019). Oleacein Prevents High Fat Diet-Induced a Diposity and Ameliorates Some Biochemical Parameters of Insulin Sensitivity in Mice. Nutrients.

[12] Lombardo G.E., Lepore S.M., Morittu V.M., Arcidiacono B., Colica C., Procopio A., Maggisano V., Bulotta S., Costa N., Mignogna C. (2018). Effects of Oleacein on High-Fat Diet-Dependent Steatosis, Weight Gain, and Insulin Resistance in Mice. Front. Endocrinol..

[13] Filipek A., Mikołajczyk T.P., Guzik T.J., Naruszewicz M. (2020). Oleacein and Foam Cell Formation in Human Monocyte-Derived Macrophages: A Potential Strategy against Early and Advanced Atherosclerotic Lesions. Pharmaceuticals.

[14] Filipek A., Czerwińska M.E., Kiss A.K., Polański J.A., Naruszewicz M. (2017). Oleacein May Inhibit Destabilization of Carotid Plaques from Hypertensive Patients. Impact on High Mobility Group Protein-1. Phytomedicine.

[15] Polini B., Digiacomo M., Carpi S., Bertini S., Gado F., Saccomanni G., Macchia M., Nieri P., Manera C., Fogli S. (2018). Oleocanthal and Oleacein Contribute to the in Vitro Therapeutic Potential of Extra Virgin Oil-Derived Extracts in Non-Melanoma Skin Cancer. Toxicol. Vitr..

[16] Cirmi S., Celano M., Lombardo G.E., Maggisano V., Procopio A., Russo D., Navarra M. (2020). Oleacein Inhibits STAT3, Activates the Apoptotic Machinery, and Exerts Anti-Metastatic Effects in the SH-SY5Y Human Neuroblastoma Cells. Food Funct..

[17] Juli G., Oliverio M., Bellizzi D., Cantafio M.E.G., Grillone K., Passarino G., Colica C., Nardi M., Rossi M., Procopio A. (2019). Anti-Tumor Activity and Epigenetic Impact of the Polyphenol Oleacein in Multiple Myeloma. Cancers.

[18] Gutiérrez-Miranda B., Gallardo I., Melliou E., Cabero I., Álvarez Y., Magiatis P., Hernández M., Nieto M.L. (2020). Oleacein Attenuates the Pathogenesis of Experimental Autoimmune Encephalomyelitis through Both Antioxidant and Anti-Inflammatory Effects. Antioxidants.

[19] Czerwińska M., Kiss A.K., Naruszewicz M. (2012). A Comparison of Antioxidant Activities of Oleuropein and Its Dialdehydic Derivative from Olive Oil, Oleacein. Food Chem..

[20] Surace A.E.A., Hedrich C.M. (2019). The Role of Epigenetics in Autoimmune/Inflammatory Disease. Front. Immunol..

[21] Montoya T., Castejón M.L., Muñoz-García R., Alarcón-De-la-Lastra C. (2021). Epigenetic Linkage of Systemic Lupus Erythematosus and Nutrition. Nutr. Res. Rev..

[22] Lauterbach M.A., Hanke J.E., Serefidou M., Mangan M.S.J., Kolbe C.C., Hess T., Rothe M., Kaiser R., Hoss F., Gehlen J. (2019). Toll-like Receptor Signaling Rewires Macrophage Metabolism and Promotes Histone Acetylation via ATP-Citrate Lyase. Immunity.

[23] Castejón M.L., Montoya T., Alarcón-De-La-Lastra C., González-Benjumea A., Vázquez-Román M.V., Sánchez-Hidalgo M. (2020). Dietary Oleuropein and Its Acyl Derivative Ameliorate Inflammatory Response in Peritoneal Macrophages from Pristane-Induced SLE Mice: Via Canonical and Noncanonical NLRP3 Inflammasomes Pathway. Food Funct..

[24] Aparicio-Soto M., Sánchez-Hidalgo M., Cárdeno A., González-Benjumea A., Fernández-Bolaños J.G., Alarcón-de-la-Lastra C. (2017). Dietary Hydroxytyrosol and Hydroxytyrosyl Acetate Supplementation Prevent Pristane-Induced Systemic Lupus Erythematous in Mice. J. Funct. Foods.

[25] Montoya T., Alarcón-De-La-Lastra C., Castejón M.L., Ortega-Vidal J., Altarejos J., Sánchez-Hidalgo M. (2022). (−)-Methyl-Oleocanthal, a New Oleocanthal Metabolite Reduces LPS-Induced Inflammatory and Oxidative Response: Molecular Signaling Pathways and Histones Epigenetic Modulation. Antioxidants.

[26] Hung Y.L., Wang S.C., Suzuki K., Fang S.H., Chen C.S., Cheng W.C., Su C.C., Yeh H.C., Tu H.P., Liu P.L. (2019). Bavachin Attenuates LPS-Induced Inflammatory Response and Inhibits the Activation of NLRP3 Inflammasome in Macrophages. Phytomedicine.

[27] Aparicio-Soto M., Sánchez-Fidalgo S., González-Benjumea A., Maya I., Fernández-Bolaños J.G., Alarcón-de-la-Lastra C. (2015). Naturally Occurring Hydroxytyrosol Derivatives: Hydroxytyrosyl Acetate and 3,4-Dihydroxyphenylglycol Modulate Inflammatory Response in Murine Peritoneal Macrophages. Potential Utility as New Dietary Supplements. J. Agric. Food Chem..

[28] Li L., Maitra U., Singh N., Gan L. (2010). Molecular Mechanism Underlying LPS-Induced Generation of Reactive Oxygen Species in Macrophages. FASEB J..

[29] Li D., Xue M., Geng Z., Chen P. (2012). Cellular Physiology Cellular Physiology Cellular Physiology Cellular Physiology The Suppressive Effects of Bursopentine (BP5) on Oxidative Stress and NF- ΚB Activation in Lipopolysaccharide-Activated Murine Peritoneal Macrophages. Cell. Physiol. Biochem..

[30] Castejon M.L., Sánchez-Hidalgo M., Aparicio-Soto M., González-Benjumea A., Fernández-Bolaños J.G., Alarcón-de-la-Lastra C. (2019). Olive Secoiridoid Oleuropein and Its Semisynthetic Acetyl-Derivatives Reduce LPS-Induced Inflammatory Response in Murine Peritoneal Macrophages via JAK-STAT and MAPKs Signaling Pathways. J. Funct. Foods.

[31] Cirmi S., Maugeri A., Russo C., Musumeci L., Navarra M., Lombardo G.E. (2022). Oleacein Attenuates Lipopolysaccharide-Induced Inflammation in THP-1-Derived Macrophages by the Inhibition of TLR4/MyD88/NF-κB Pathway. Int. J. Mol. Sci..

[32] Castejón M.L., Montoya T., Alarcón-de-la-lastra C., Sánchez-hidalgo M. (2020). Potential Protective Role Exerted by Secoiridoids from Olea Europaea l. In Cancer, Cardiovascular, Neurodegenerative, Aging-Related, and Immunoinflammatory Diseases. Antioxidants.

[33] Cuevas B., Arroba A.I., de los Reyes C., Gómez-Jaramillo L., González-Montelongo M.C., Zubía E. (2021). Diterpenoids from the Brown Alga Rugulopteryx Okamurae and Their Anti-Inflammatory Activity. Mar. Drugs.

[34] Tang H., Roy P., Di Q., Ma X., Xiao Y., Wu Z., Quan J., Zhao J., Xiao W., Chen W. (2022). Synthesis Compound XCR-7a Ameliorates LPS-Induced Inflammatory Response by Inhibiting the Phosphorylation of c-Fos. Biomed. Pharmacother..

[35] Montoya T., Castejón M.L., Sánchez-Hidalgo M., González-Benjumea A., Fernández-Bolaños J.G., Alarcón De-La-Lastra C. (2019). Oleocanthal Modulates LPS-Induced Murine Peritoneal Macrophages Activation via Regulation of Inflammasome, Nrf-2/HO-1, and MAPKs Signaling Pathways. J. Agric. Food Chem..

[36] Montoya T., Aparicio-Soto M., Castejón M.L., Rosillo M.Á., Sánchez-Hidalgo M., Begines P., Fernández-Bolaños J.G., Alarcón-de-la-Lastra C. (2018). Peracetylated Hydroxytyrosol, a New Hydroxytyrosol Derivate, Attenuates LPS-Induced Inflammatory Response in Murine Peritoneal Macrophages via Regulation of Non-Canonical Inflammasome, Nrf2/HO1 and JAK/STAT Signaling Pathways. J. Nutr. Biochem..

[37] Rosignoli P., Fuccelli R., Fabiani R., Servili M., Morozzi G. (2013). Effect of Olive Oil Phenols on the Production of Inflammatory Mediators in Freshly Isolated Human Monocytes. J. Nutr. Biochem..

[38] Cargnello M., Roux P.P. (2011). Activation and Function of the MAPKs and Their Substrates, the MAPK-Activated Protein Kinases. Microbiol. Mol. Biol. Rev..

[39] Arthur J.S.C., Ley S.C. (2013). Mitogen-Activated Protein Kinases in Innate Immunity. Nat. Rev. Immunol..

[40] Berköz M. (2019). Diosmin Suppresses the Proinflammatory Mediators in Lipopolysaccharide-Induced RAW264.7 Macrophages via NF-ΚB and MAPKs Signal Pathways. Gen. Physiol. Biophys..

[41] Wang Z., Zhang S., Xiao Y., Zhang W., Wu S., Qin T., Yue Y., Qian W., Li L. (2020). NLRP3 Inflammasome and Inflammatory Diseases. Oxid. Med. Cell. Longev..

[42] Zheng D., Liwinski T., Elinav E. (2020). Inflammasome Activation and Regulation: Toward a Better Understanding of Complex Mechanisms. Cell Discov..

[43] Matikainen S., Nyman T.A., Cypryk W. (2020). Function and Regulation of Noncanonical Caspase-4/5/11 Inflammasome. J. Immunol..

[44] Hedrich C.M. (2018). Mechanistic Aspects of Epigenetic Dysregulation in SLE. Clin. Immunol..

[45] Stefanowicz D., Lee J.Y., Lee K., Shaheen F., Koo H.K., Booth S., Knight D.A., Hackett T.L. (2015). Elevated H3K18 Acetylation in Airway Epithelial Cells of Asthmatic Subjects. Respir. Res..

[46] Imuta H., Fujita D., Oba S., Kiyosue A., Nishimatsu H., Yudo K., Suzuki E. (2020). Histone Methylation and Demethylation Are Implicated in the Transient and Sustained Activation of the Interleukin-1β Gene in Murine Macrophages. Heart Vessel..

[47] Zhao S., Zhong Y., Fu X., Wang Y., Ye P., Cai J., Liu Y., Sun J., Mei Z., Jiang Y. (2019). H3K4 Methylation Regulates LPS-Induced Proinflammatory Cytokine Expression and Release in Macrophages. Shock.

[48] Yi Y.S. (2021). Functional Interplay between Methyltransferases and Inflammasomes in Inflammatory Responses and Diseases. Int. J. Mol. Sci..

[49] Sun H.J., Ren X.S., Xiong X.Q., Chen Y.Z., Zhao M.X., Wang J.J., Zhou Y.B., Han Y., Chen Q., Li Y.H. (2017). Nlrp3 Inflammasome Activation Contributes to Vsmc Phenotypic Transformation and Proliferation in Hypertension. Cell Death Dis..

[50] Corominas-Faja B., Cuyàs E., Lozano-Sánchez J., Cufí S., Verdura S., Fernández-Arroyo S., Borrás-Linares I., Martin-Castillo B., Martin G., Lupu R. (2018). Extra-Virgin Olive Oil Contains a Metabolo-Epigenetic Inhibitor of Cancer Stem Cells. Carcinogenesis.

[51] Cuyàs E., Verdura S., Menendez J.A., Carreras D., Verdura S., Brugada R., Bosch-Barrera J., Gumuzio J., Martin Á.G., Lozano-Sánchez J. (2019). Extra Virgin Olive Oil Contains a Phenolic Inhibitor of the Histone Demethylase LSD1/KDM1A. Nutrients.

[52] Carpi S., Polini B., Manera C., Digiacomo M., Salsano J.E., Macchia M., Scoditti E., Nieri P. (2020). MiRNA Modulation and Antitumor Activity by the Extra-Virgin Olive Oil Polyphenol Oleacein in Human Melanoma Cells. Front. Pharmacol..

[53] Carpi S., Scoditti E., Massaro M., Polini B., Manera C., Digiacomo M., Salsano J.E., Poli G., Tuccinardi T., Doccini S. (2019). The Extra-Virgin Olive Oil Polyphenols Oleocanthal and Oleacein Counteract Inflammation-Related Gene and Mirna Expression in Adipocytes by Attenuating Nf-Κb Activation. Nutrients.

[54] Diez-Bello R., Jardin I., Lopez J.J., El Haouari M., Ortega-Vidal J., Altarejos J., Salido G.M., Salido S., Rosado J.A. (2019). (−)-Oleocanthal Inhibits Proliferation and Migration by Modulating Ca^2+^ Entry through TRPC6 in Breast Cancer Cells. Biochim. Biophys. Acta-Mol. Cell Res..

[55] Vougogiannopoulou K., Lemus C., Halabalaki M., Pergola C., Werz O., Smith A.B., Michel S., Skaltsounis L., Deguin B. (2014). One-Step Semisynthesis of Oleacein and the Determination as a 5-Lipoxygenase Inhibitor. J. Nat. Prod..

[56] Montserrat-De La Paz S., García-Giménez M.D., Ángel-Martín M., Pérez-Camino M.C., Fernández Arche A. (2014). Long-Chain Fatty Alcohols from Evening Primrose Oil Inhibit the Inflammatory Response in Murine Peritoneal Macrophages. J. Ethnopharmacol..

[57] Re R., Pellegrini N., Proteggente A., Pannala A., Yang M., Rice-Evans C. (1999). Antioxidant Activity Applying an Improved ABTS Radical Cation Decolorization Assay. Free Radic. Biol. Med..

[58] Hajji N., Joseph B. (2010). Epigenetic Regulation of Cell Life and Death Decisions and Deregulation in Cancer. Essays Biochem..

[59] Bradford M.M. (1976). A Rapid and Sensitive Method for the Quantitation of Microgram Quantities of Protein Utilizing the Principle of Protein-Dye Binding. Anal. Biochem..

