# Supplementary material for: Effects of Oleacein, a New Epinutraceutical Bioproduct from Extra Virgin Olive Oil, in LPS-Activated Murine Immune Cells
Source: Pharmaceuticals (Basel). 2022 Oct 28;15(11):1338. doi: 10.3390/ph15111338 (PMC9699377; doi:10.3390/ph15111338)
Supplement: Supplementary file 1 [file pharmaceuticals-15-01338-s001.zip › Supplementary material.pdf]

Supplementary Files

Effects of Oleacein, a New Epinutraceutical Bioproduct from Extra-Virgin Olive Oil, in LPS-Activated Murine Immune Cells.

Rocío Muñoz-García[1,2], Marina Sánchez-Hidalgo[1,2], Tatiana Montoya[1,2], Manuel Alcarranza[1,2], Juan Ortega-Vidal[3], Joaquín Altarejos[3], Catalina Alarcón-de-la-Lastra*[1,2].

1. Department of Pharmacology, Faculty of Pharmacy, Universidad de Sevilla, 41012 Sevilla, Spain
2. Instituto de Biomedicina de Sevilla, IBiS (Universidad de Sevilla, HUVR, Junta de Andalucía, CSIC), Seville, Spain
3. Department of Inorganic and Organic Chemistry, Faculty of Experimental Sciences, Campus de Excelencia Internacional Agroalimentario (ceiA3), Universidad de Jaén, 23071 Jaén, Spain

*Correspondence: calarcon@us.es; Tel.: +34954559877

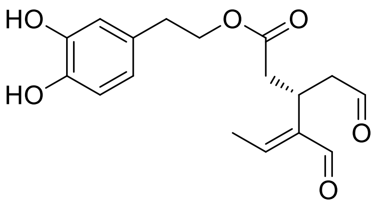

(-)-Oleacein (OLA)

Figure S1. Oleacein chemical structure

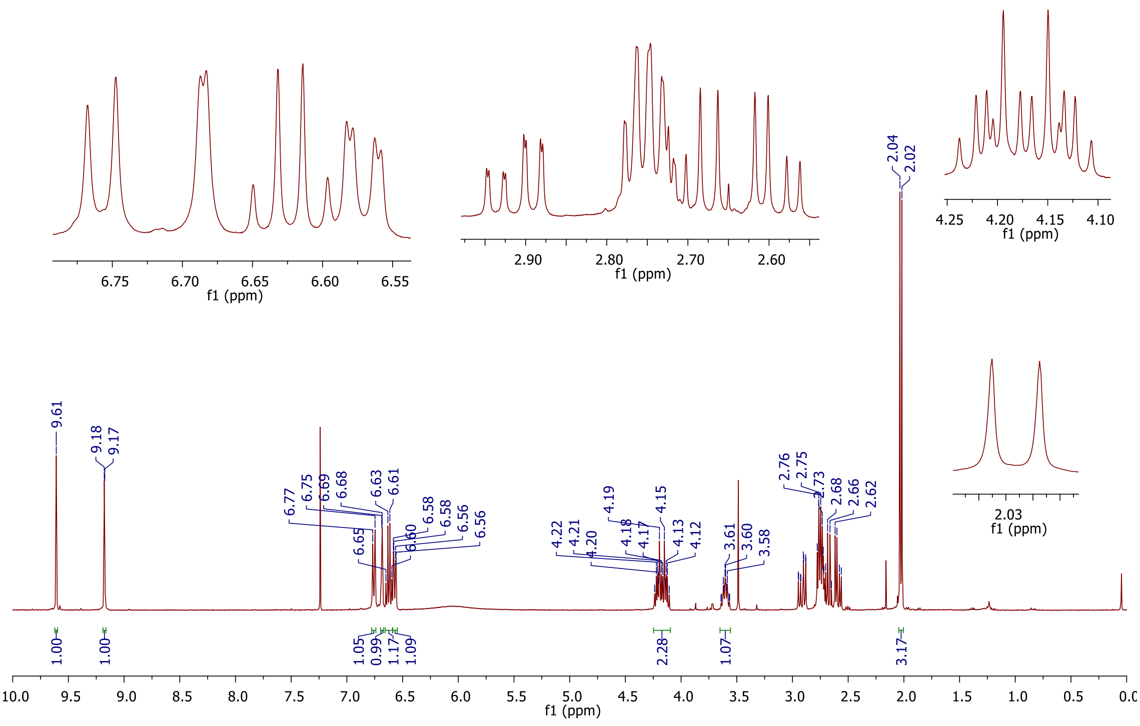

Figure S2. ^{1}H NMR of (–)-Oleacein in deuterated chloroform.

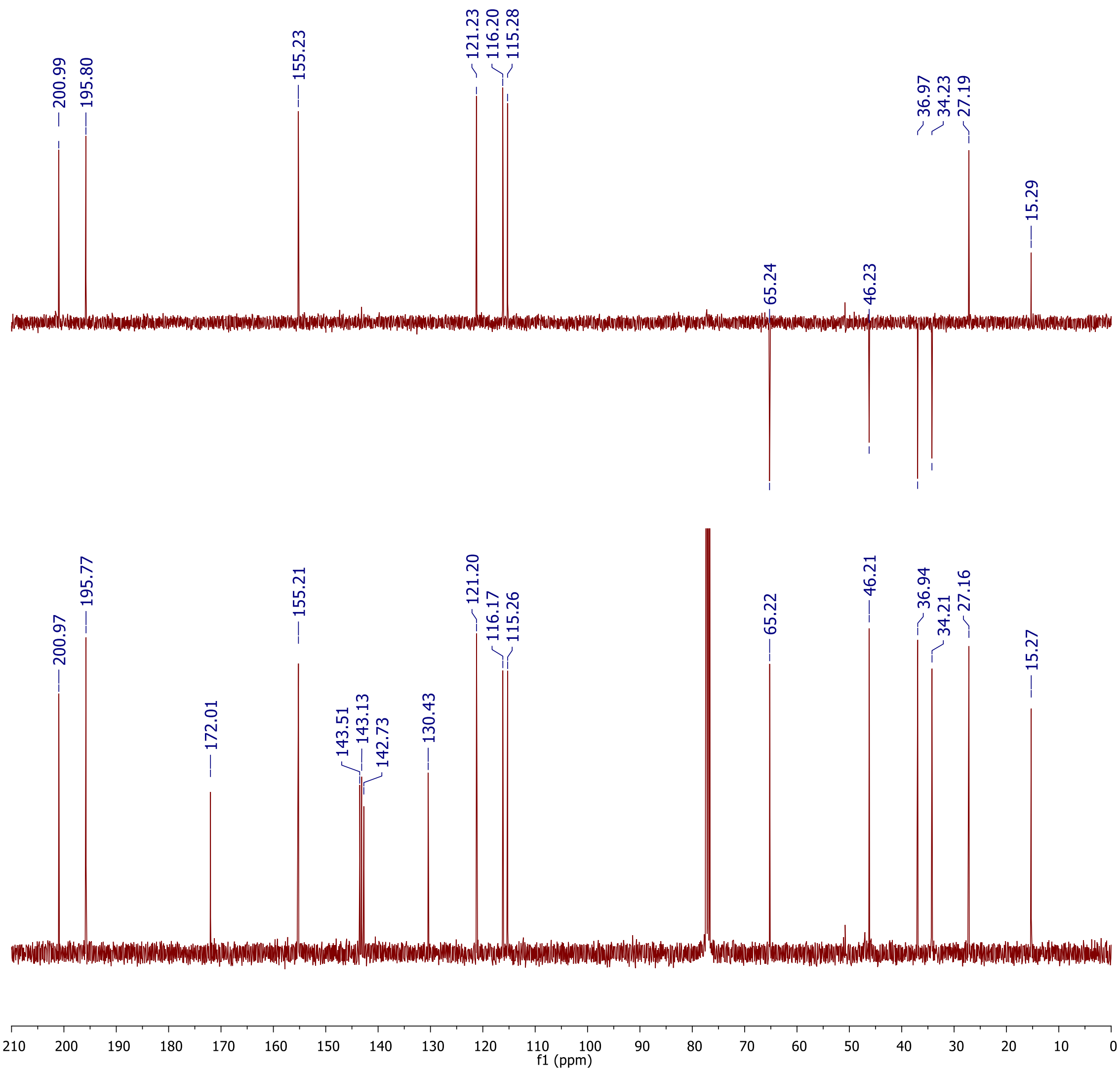

Figure S3. ^{13}C NMR (bottom) and Dept-135 (superior) of (–)-Oleacein in deuterated chloroform.
