# Supplementary figures and images for: Effects of Oleacein, a New Epinutraceutical Bioproduct from Extra Virgin Olive Oil, in LPS-Activated Murine Immune Cells
Source: Pharmaceuticals (Basel). 2022 Oct 28;15(11):1338. doi: 10.3390/ph15111338 (PMC9699377; doi:10.3390/ph15111338)

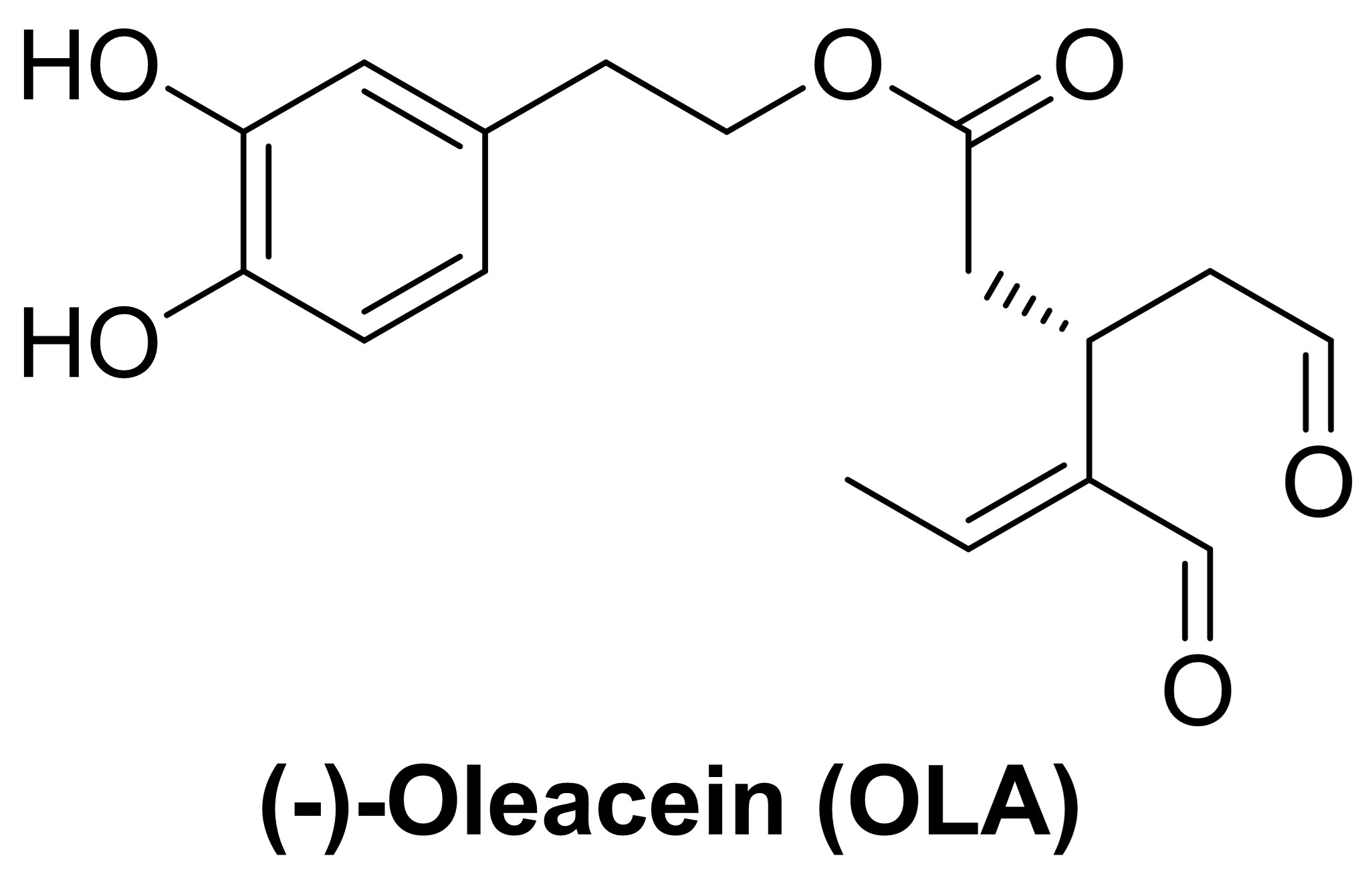

Supplement: Supplementary file 1 [file pharmaceuticals-15-01338-s001.zip › Figure S1.png]

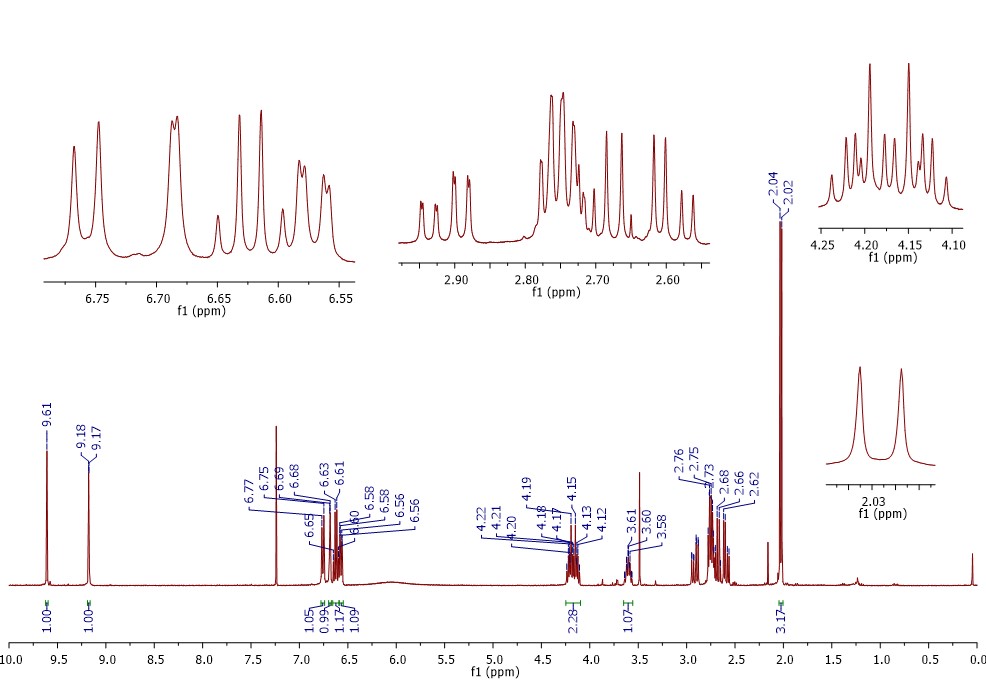

Supplement: Supplementary file 1 [file pharmaceuticals-15-01338-s001.zip › Figure S2.png]

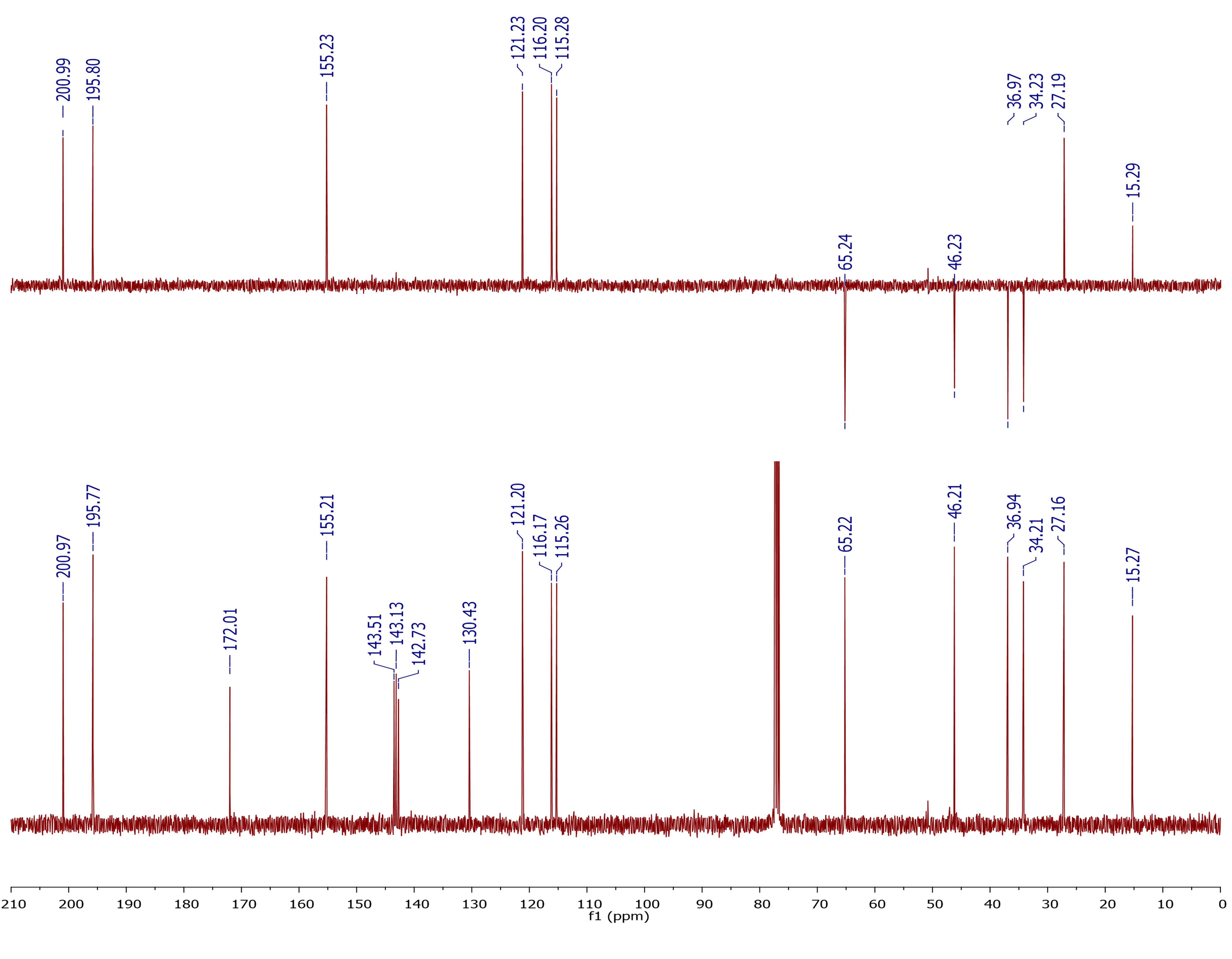

Supplement: Supplementary file 1 [file pharmaceuticals-15-01338-s001.zip › Figure S3.png]
